# A stepped-care model of post-disaster child and adolescent mental health service provision

**DOI:** 10.3402/ejpt.v5.24294

**Published:** 2014-07-11

**Authors:** Brett M. McDermott, Vanessa E. Cobham

**Affiliations:** 1 Kids in Mind Research, Mater Research, University of Queensland, Brisbane, Australia; 2 Department of Psychology, University of Queensland, Brisbane, Australia

**Keywords:** Disaster planning, mental health services, child, adolescent

## Abstract

**Background:**

From a global perspective, natural disasters are common events. Published research highlights that a significant minority of exposed children and adolescents develop disaster-related mental health syndromes and associated functional impairment. Consistent with the considerable unmet need of children and adolescents with regard to psychopathology, there is strong evidence that many children and adolescents with post-disaster mental health presentations are not receiving adequate interventions.

**Objective:**

To critique existing child and adolescent mental health services (CAMHS) models of care and the capacity of such models to deal with any post-disaster surge in clinical demand. Further, to detail an innovative service response; a child and adolescent stepped-care service provision model.

**Method:**

A narrative review of traditional CAMHS is presented. Important elements of a disaster response – individual versus community recovery, public health approaches, capacity for promotion and prevention and service reach are discussed and compared with the CAMHS approach.

**Results:**

Difficulties with traditional models of care are highlighted across all levels of intervention; from the ability to provide preventative initiatives to the capacity to provide intense specialised posttraumatic stress disorder interventions. In response, our over-arching stepped-care model is advocated. The general response is discussed and details of the three tiers of the model are provided: Tier 1 communication strategy, Tier 2 parent effectiveness and teacher training, and Tier 3 screening linked to trauma-focused cognitive behavioural therapy.

**Conclusion:**

In this paper, we argue that traditional CAMHS are not an appropriate model of care to meet the clinical needs of this group in the post-disaster setting. We conclude with suggestions how improved post-disaster child and adolescent mental health outcomes can be achieved by applying an innovative service approach.

In this paper, we will argue that following exposure to a single event trauma such as a natural disaster, a significant minority of children will develop a mental health presentation that would benefit from a clinical intervention. However, given that clinical services typically operate at capacity at any given point in time, existing service systems cannot be expected to meet the post-disaster surge in demand. Further, we argue that existing clinical care models focus on severe and complex presentations, are expensive, and offer little to mild and moderately affected individuals. We will detail our solution; a stepped-care service model that encompasses both community recovery and individual treatment. We will explain how this stepped-care approach facilitates efficient use of limited post-disaster financial resources whilst maximising the reach of interventions. Evidence regarding the specific interventions discussed will be cited.

## Burden of child and youth mental health care following a natural disaster

There is now a substantial literature on child and adolescent post-disaster mental health presentations. Precise estimates of adverse outcomes are not possible given disasters vary greatly in terms of loss of life and exposure to life-threatening experiences. Further, existing research varies in the measures used, sample demographics, and the amount of time between the disaster and data collection. From a general psychopathology perspective, 9.3% of 4–17-year-old children were described as experiencing a “serious emotional disturbance” 18–27 months after Hurricane Katrina (McLaughlin et al., 2009). This is consistent with large sample cross-sectional research following a range of different disasters that has reported approximately 5–15% of children experience significant mental health symptoms following disaster exposure (McDermott & Palmer, 1999; Roussos et al., 2005; Shaw, Applegate, & Schorr, 1996; Thienkrua, Cardozo, Chakkraband, Guadamuz, & Thailand Post-Tsunami Mental Health Study Group, 2006; Vernberg, Silverman, La Greca, & Prinstein, 1996). Posttraumatic stress (PTS) symptoms have been frequently reported: 6 and 57% of children exposed to the 2004 Tsunami disaster experienced significant PTS symptoms (Thienkrua et al., 2006; Wickrama & Kaspar, 2007); 35% of children exposed to hurricanes (La Greca, Silverman, Lai, & Jaccard, 2010) and 4.5–95% of children exposed to earthquakes (Eksi & Braun, 2009; Roussos et al., 2005). Presentations other than PTS include depression and anxiety. In a large adolescent sample reviewed 6 months after an earthquake in China, 24.5% reported clinically significant depressive symptoms and 40.5% reported clinically significant anxiety symptoms. Similar rates of depressive symptoms and anxiety symptoms have been reported after a wildfire in Greece (Papadatou et al., 2012), and somewhat lower rates, that is, 17.6 and 12%, respectively (consistent with data collection, 14 months post-disaster), have been reported following a cyclone in India (Kar & Bastia, 2006). Other posttraumatic presentations include elevated rates of substance abuse (Reijneveld, Crone, Verhulst, & Verloove-Vanhorick, 2003), somatic symptoms (Hensley & Varela, 2008), and aggression (Scheeringa & Zeanah, 2008).

Symptom chronicity in primary school children is common, with reports of moderate to severe levels of PTS symptoms ranging from 20% 18 months after a cyclone (McDermott, Cobham, Berry, & Kim, 2014) to 29% 21 months after a hurricane (Shaw et al., 1996). Focusing on post-disaster psychiatric diagnoses, one-third of primary school children still met Diagnostic and Statistical Manual 4th Edition (DSM-IV) criteria for a mental illness 36 months after surviving the 2006 tsunami (Ularntinon et al., 2008). From a service utilisation perspective, 3 years after Hurricane Katrina, 27.7% of children met criteria for a mental health referral (Kronenberg et al., 2010). Contemporary understanding of adverse post-disaster outcomes includes evidence of functional impairment such as sub-optimal academic performance and school drop-out (Corliss, Lawrence, & Nelson, 2008), more school absence (Broberg, Dyregov, & Lilled, 2005), and family and peer relationship difficulties (Satcher, 2000). Studies have reported that parental distress, including parent PTS symptoms, is associated with children's post-disaster levels of functioning (Kilic, Kilic, & Aydin, 2011; Scheeringa & Zeanah, 2008). The mechanism of these relationships remains unclear; however, they may include alterations in family communication (Kilic et al., 2011; Scheeringa & Zeanah, 2001), the development of unhelpful parenting practices (Scaramella, Sohr-Preston, Callahan, & Mirabile, 2008), and over-protectiveness leading to decreased exposure to feared situations and usual social activities (Dyb, Jensen, & Nygaard, 2011).

Finally, there is increasing evidence linking the experience of emotional trauma to diminished quality of life. Alisic and colleagues reported a significant correlation between posttraumatic stress reactions across a broad range of exposures and diminished quality of life in a community sample of primary school children (Alisic, van der Shoot, van Ginkel, & Kleber, 2008). PTS symptoms in children 1 month following a motor vehicle accident were predictive of health-related quality of life (HRQOL) 1 year following the accident (Landolt, Vollrath, Gnehm, & Sennhauser, 2009). A similar relationship, post-burn PTS symptoms and later decreased HRQOL, remained significant after controlling for size of burn, duration of hospitalisation, and number of surgical procedures (Landolt, Buehlmann, Maag, & Schiestl, 2009). Song and colleagues demonstrated that in primary school, children who witnessed the accidental death of two mothers during a school fire drill, children with depressive symptoms at 6 months were statistically associated with lower parent ratings of child HRQOL at 30 months (Song et al., 2012). The latter finding replicated Goenjian and colleagues who reported depression at 3 months was the strongest predictor of HRQOL at 32 months in adolescents who were exposed to a Greek earthquake (2011).

## Service delivery in the post-disaster setting

Child and adolescent mental health services (CAMHS) are typically public-funded therapy services organised within government departments of health. Most are multi-disciplinary employing a range of professionals who provide family therapy, individual counselling, or psychopharmacology treatments. Therapy is often delivered in community clinics. Better-resourced services have access to inpatient teams and partial hospitalisation programmes. Generally, CAMHS are secondary or tertiary services in that they receive referrals from general practitioners, school counsellors, or paediatricians. Referrals from a hospital accident and emergency department are common. CAMHS capacity often varies across country, state, and region. Some countries are also well-endowed with a broad range of other emotional health services including school-based counsellors and chaplaincy services and non-government organisations (NGOs) that can provide direct, telephone, or online services. Private health care systems are important additions to capacity in some countries’; however, often have very limited reach.

In countries with more limited investment in child and youth mental health, services range from no dedicated service to a modest intervention capacity. Examples of the latter include services with only child psychiatrists and limited clinical child psychology, social work, or child therapy capacity. Even from the perspective of a high investment country, current CAMHS models of care cannot meet the existing community burden of illness. In the Australian context, only 1 in 5 to 1 in 7 of those who may benefit actually receive a service (Sawyer et al., 2001). Similarly, of a representative school sample of 1,035 German adolescents aged 12–17 years, 192 young people met diagnostic criteria for an anxiety disorder. Of these, only 35 (18.2%) received mental health treatment (Essau, Conradt, & Petermann, 2002).

Work in the disaster field has major differences to the typical CAMHS service delivery models. Some differences are obvious, for example, closure of roads and public services due to building damage and public transport disruption. Equally important are differences in the usual emotional health care pathways. Often parents and children who develop post-disaster syndromes have no tradition of accessing mental health services. They may also have strong views about the stigma associated with acknowledging a mental health, emotional, or behaviour problem (Stevens, Kelleher, Ward-Estes, & Ward, 2006). Assessment issues include clinicians favouring parent report of children's mental health condition (Grills & Ollendick, 2003), even though it is the child's self-report that is crucial to reaching a posttraumatic stress disorder (PTSD) diagnosis, the low parent–child agreement for the presence of child acute stress disorder (Kassam-Adams, García-España, Miller, & Winston, 2006) and low parent–child correlation of the number of traumatic events experienced and their impact (Meiser-Stedman, Smith, Glucksman, Yule, & Dagleish, 2007). Parent identification of child symptoms is made more difficult given the empirical evidence that some children purposely withhold details of their posttraumatic symptoms in the belief their parents have enough to cope with in the post-event environment (Stallard & Law, 1993). Finally, parents who themselves are experiencing grief or posttraumatic mental health symptoms may also be less proficient at identifying children with emotional problems.

A ubiquitous post-disaster service provision challenge is the potential for new referrals and aligning this with service surge capacity. Surge capacity (the ability to deal with a sudden influx of new work) following weather-related natural disasters has been described as poor, even in well-resourced health systems such as Australia (Blashki, Berry, & Kidd, 2009). Other contextual issues include the observation that local mental health leaders may themself be impacted through exposure to the event, material or personal losses or they may be experiencing symptoms of posttraumatic stress. These circumstances represent some of the barriers that often prevent local clinical leaders from quickly up-skilling in the area of disaster mental health and developing an integrated plan. Note a temporary reorientation towards treating disaster-affected clients is not a plausible service solution given it is both unethical and impractical for services to stop providing treatment to their existing clientele. The service provision ethos outlined in this paper is based on recent research and the authors’ experience that following a natural disaster the typical service provision conundrum is: (1) there is extensive empirical data on service need (prevalence of post-traumatic mental health presentations), indeed there is a contemporary rapid needs assessment methodology to clarify this issue (Dalton, Scheeringa, & Zeanah, 2008), and illness burden (symptom chronicity, functional impairment, and lowered quality of life), (2) many individuals in the disaster-affected community will have a high level of concern about children and will often ask what counselling services are doing to address children's needs, and (3) few children and families will present for care (Fairbrother, Stuber, Galea, Pfefferbaum, & Fleischman, 2004; Geddie Pullins, McCammon, Smith Lamson, Wuensch, & Mega, 2005; see discussion by Scheeringa, Cobham, & McDermott (2014 )on service uptake following the World Trade Center disaster and following Hurricane Katrina). We advocate for a very proactive approach. Thus, we actively involve authorities and systems (e.g., education) that have access to children and families, and employ strategies that are more assertive forms of clinical case identification. However, one crucial ethical issue is the availability of post-disaster treatment resources. A proactive assertive approach is not appropriate if it is highly unlikely treatment resources can be mobilised to service the identified children or if the magnitude of the disaster is such that an individual or family service response is impractical. For example, given the loss of life and devastation following the 2011 Japanese Tsunami, a case identification screen for post-disaster mental health disorders would identify a need that far exceeded the resources available for treatment.

## Stepped-care approaches

Some service providers and clinicians have argued a graduated service response that starts with less intensive interventions may be more effective in reducing symptoms, impairment, and improving the quality of life of individuals with mental health problems (Marks, 2010). Bower and Gilbody outline the assumptions that underpin a stepped-care approach (2005). The equivalence assumption suggests for some clinical presentations the gains from low-intensity interventions equal the gains from traditional psychotherapy. The efficiency assumption suggests that minimal intervention therapies are a more cost effective use of resources. The accessibility assumption suggests that minimal intervention therapies are acceptable to both patients and professionals. The key features of stepped-care models are starting with a low-intensity intervention, monitoring to establish treatment benefits or lack of response, and having the capability to step up to a higher intensity treatment (Seekles, van Straten, Beekman, van Marwijk, & Cuijpers, 2009). Van Straten, Seekles, Van't Veer-tazelaar, Beekman, and Cuijpers (2010) advise that minimal delay in progressing to the next level is important and have suggested that the duration of treatment per level is limited (6–12 weeks). The stepped-care models cited in published clinical practice guidelines generally take the form of level 1 being “watchful waiting”; level 2 is guided self-help plus or minus support to prevent patient drop-out; level 3 is face-to-face brief therapy; and level 4 is longer term therapy, medication or a combination of both (National Collaborating Centre for Mental Health, 2005).

There are two other important considerations that underpin the stepped-care philosophy. First, there is an emphasis to include interventions that have an established evidence base. The second is the poor reach of current treatments for emotional and behavioural disorders of childhood, and the capacity for a stepped-care model to overcome this. For example, the treatment of childhood anxiety disorders; despite increasing evidence of effective interventions, most anxiety-disordered children do not receive treatment (Essau et al., 2002).

One way of potentially increasing reach is to offer more brief interventions. A one-session cognitive behaviour therapy (CBT) approach for children and adults with specific phobias has been successfully trialled (Ollendick et al., 2009; Ost, 1997) but has not been investigated with posttraumatic presentations. Increased reach may also be achieved with interventions that have a smaller time burden on busy parents and caregivers. Sanders has argued that parenting programmes, often delivered in seminar format and outside of usual working hours, can greatly increase intervention reach (Sanders, 2012). An alternative approach to increase the reach of a given intervention is a more comprehensive change in the way services are delivered. Zatzick and colleagues studied the population impact of interventions by comparing a stepped collaborative care model (CC) and a CBT intervention in adults with significant PTSD and/or depression following an injury (Zatzick, Koepsell, & Rivara, 2009). The CBT intervention (4–6, 60–90 min sessions) resulted in a large treatment effects size. However, given the range of intervention-specific exclusions, the estimated intervention reach was only 27/10000. The CC approach (case management, motivational interviewing, CBT, psychopharmacological or combined interventions as preferred by the patient) had a much smaller effect size; however, the reach was substantially greater (1762/10000). The authors concluded CC led to a 9.5-fold greater impact on PTSD prevention (Zatzick et al., 2009).

## The Queensland stepped-care model employed after the 2010/2011 flood disaster

**Fig. 1:**
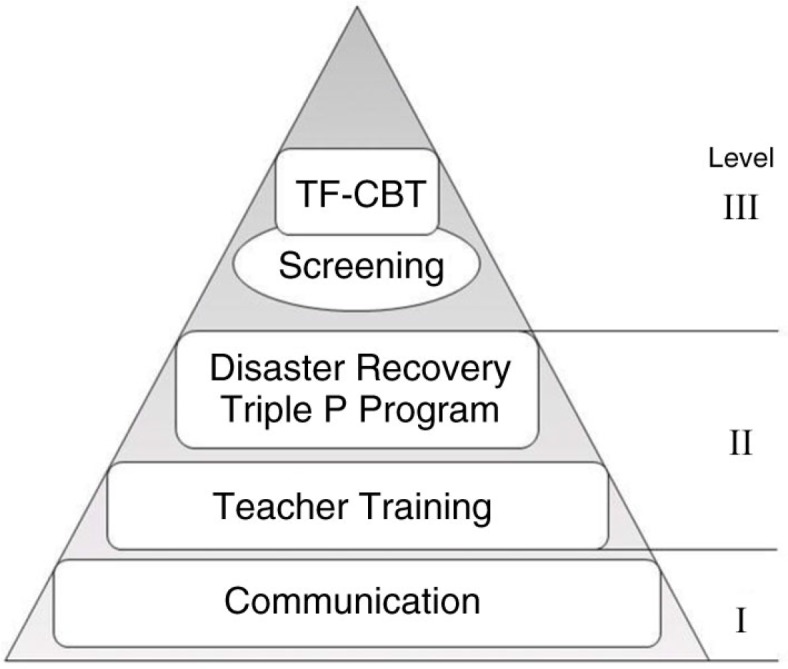
The Queensland 2011 disaster stepped-care service provision model for children, adolescents, and families.

During the summer months of 2010/2011, Queensland experienced a major flood disaster. Over half of Queensland was affected including the capital city and 70 towns; approximately 200,000 individuals were either evacuated or displaced and tragically 35 individuals died. Damage to public infrastructure was estimated at over $AUD 6 billion (PricewaterhouseCoopers [PwC], 2011). Figure 1 details the stepped-care model developed by the authors, the chair and deputy-chair of the government funded child and youth mental health response. Initiatives closer to the base of the triangle are low intensity, large reach. Initiatives closer to the apex require greater clinician training, are more costly to deliver, more intensive, and are offered to children, adolescents, and families with more complex and severe presentations. The lowest-intensity response was a communication strategy that occurred during the watchful waiting period. Communication interventions included information pod-casts on the Education Queensland website and on YouTube, and dissemination of information pamphlets and resources available online such as those available through the Australian Early Trauma Loss Network (www.earlytraumagrief.org.au). More intensive communication undertakings included community consultation forums offered in disaster-affected communities, which were open to any concerned parent, teacher, counsellor, or other adult who wished to attend. These forums were delivered in local schools, community halls, or designated disaster recovery centres.

Level 2 of the stepped-care model included up-skilling professionals to deliver a specially developed parenting intervention and providing training to teachers. Teacher training took the form of a 2–3 hour one-off seminar (Kenardy, De Young, Le Brocque, & March, 2011). The training programme provided teachers with an understanding of posttraumatic mental health syndromes in children, how they can be conceptualised as a deviation from the child's pre-disaster developmental trajectory, how these syndromes might be manifested in the classroom and referral pathways for children in need of a mental health assessment. The seminar did not seek to encourage or up-skill teachers to provide a low-intensity mental health intervention. This latter approach may be appropriate in some countries that do not have a robust pre-disaster CAMHS system of care. Indeed in many countries, teachers are the only available workforce with the appropriate developmental skills to deliver a post-disaster emotional health intervention. In such cases, candidates for teacher-delivered interventions include the ERASE-stress (Gelkopf & Berger, 2009) or KIDNET (Ruf et al., 2010) programmes, or the intervention of Wolmer, Hamiel, and Laor (2011)—see discussion in the later section on level 3 interventions. Given the Australian mental health system is relatively well-resourced, there was no need, nor political support, for teachers to deliver interventions. Teachers were strongly encouraged to promote re-establishing children's routines by focusing on teaching. Mental health practitioners provided in-reach to schools. Teachers were also provided information about mental health self-care. In the post-disaster setting, teachers are exposed to their students’ distress and possible dysregulation over a protracted period of time and doing so has the potential to adversely affect their own mental health. The programme presented the opportunity for indicated prevention with teachers as well as resilience building, raising awareness of mental health problems, and improving their knowledge of pathways to obtain assistance.

The other level 2 intervention was Disaster Recovery Triple P (DRTP, Cobham, McDermott, & Sanders, 2011). DRTP was developed as a collaborative initiative between the disaster recovery service and the University of Queensland Parenting and Family Support Centre. The latter are responsible for the development of Triple P (the Positive Parenting Programme) and facilitating the substantial evidence of benefit of this intervention (Sanders, 2012). DRTP is a moderately intense intervention given the format is a 2-hour parent seminar. The programme is a universal intervention given it was open to all parents who wished to attend. DRTP has the potential for considerable reach in that it can be delivered to 40 or more parents at the one time. DRTP was developed because of the emerging evidence of challenges to normal family functioning following natural disasters (Kelley et al., 2010; McDermott, Berry, & Cobham, 2012; Scaramella et al., 2008), as well as the empirical evidence of parents reporting increased over-protectiveness (Dyb et al., 2011), increased vigilance of their child, and a decrease in their willingness to facilitate child autonomy following a natural disaster (Cobham & McDermott, 2014). The seminar included psycho-education about post-disaster child presentations, parents looking after their own health, and avoiding “parenting traps” (e.g., persistent over-protection which diminishes a child's chance of symptom resolution due to normal re-exposure). Dissemination of DRTP was linked to local schools; advertising DRTP in newsletters, and delivering DRTP at the end of the school day or in the evening. Other dissemination options include linkage of DRTP with local government, through public health or general practice/family medicine opportunities. DRTP was developed to be delivered during the early (1–3 month) recovery period, subject to the availability of suitable infrastructure.

The third and highest level of our model linked the results of proactive screening to identify individuals with post-disaster mental health presentations, with offering identified children and adolescents Trauma-Focussed Cognitive Behaviour Therapy (TF-CBT). Screening has been a feature of our intervention ethos across bushfire, cyclone, and flood disasters (McDermott & Cobham, 2012). Across five Australian natural disasters, using a range of screening instruments, a substantial minority (8–12%) of students’ self-reported severe or very severe PTSD symptoms that would warrant further assessment to exclude a mental health diagnosis (McDermott & Cobham, 2012). Our experience is that school-based screening has a high level of acceptability with school principals, teachers, and parents, as well as empirical evidence of teacher and parent satisfaction (submitted manuscript). Further, seeking parent consent for screening and subsequently discussing the screening results engages parents in conversations about their child's emotional wellbeing after the traumatic event. Over time, our screening process has evolved into a two-stage procedure. The stage 1 self-report questionnaire now includes a measure of depression and anxiety along with a PTSD measure. Individuals who score above a cut-off on the stage I screen, with further parent consent, undertake both the child and parent version of a semi-structured diagnostic interview to clarify whether or not the child meets criteria for a posttraumatic mental health disorder.

The highest level of our stepped-care model is TF-CBT. Silverman and colleagues reviewed psychological treatments for youth exposed to traumatic events and concluded TF-CBT was the only treatment to meet well-established criteria for an evidenced based intervention (Silverman et al., 2008). The evidence base for TF-CBT has continued to develop including individual approaches with a focus on cognitive restructuring (Rosenberg, Jankowski, Fortuna, Rosenberg, & Mueser, 2011). Group approaches include a study of US grade 6 to 8 students exposed to “severe violence in the past year” who were randomised to 10 session group TF-CBT (SSET: “Support for students exposed to trauma” programme) or wait-list control. The authors concluded SSET contributed to a small reduction in PTSD symptoms severity (Jaycox et al., 2010). Another TF-CBT group programme (CBITS: “Cognitive behavioural intervention for trauma in schools”), also a 10 session intervention and trialled with a similar student sample, resulted in lower child- and parent-reported trauma-related symptoms (Stein et al., 2003). The most published and established version of TF-CBT is the intervention developed by Cohen, Mannarino, and Deblinger (2006) for use with youth who have experienced childhood sexual abuse. This programme has been manualised, widely researched, and disseminated internationally. The intervention is a highly structured programme in which parents and children are seen conjointly in 90-min weekly sessions. The trained clinician works through eight standard components with the parent and child.

The published effectiveness of CBT delivered in individual (Taylor & Weems, 2011) and group format (Giannopoulou, Dikaiakou, & Yule, 2006; Jaycox et al., 2010; Stein et al., 2003) has resulted in this treatment modality being the advised treatment of choice of many treatment guidelines and empirical reviews (e.g., American Academy of Child and Adolescent Psychiatry [AACAP] (2010); Australian Centre for Posttraumatic Mental Health (2013)). A new development is interventions that advocate combining CBT and narrative approaches. Salloum and Overstreet reported both group and individual approaches were effective in their manualised intervention with 7-12-year-olds 4 months after a hurricane (2008). The ERASE (“Extended enhancing resilience among students experiencing stress”) programme is a manualised 12–16 session teacher-delivered programme inclusive of psycho-education, skills, and resiliency training programme (many elements found in CBT programmes). Applying quasi-randomised controlled designs, compared to wait-list controls participation in the ERASE treatment resulted in significant PTSD symptom reduction in Sri Lankan students exposed to the 2004 Tsunami (Berger & Gelkopf, 2009), and in Israeli students exposed to terrorist attacks (Gelkopf & Berger, 2009). There is also emerging evidence that teacher-based interventions that deliver resilience-enhancing programmes prior to a trauma event result in fewer post-event posttraumatic stress and mood symptoms (Wolmer et al., 2011). There is also emerging evidence that the school-based intervention delivery approach also decreases the treatment drop-out rate. Jaycox and colleagues reported 91% of participants in the school-based condition of their trial completed therapy compared to 15% of the clinic-based condition (2010).

It should also be noted that alternatives to TF-CBT are being actively evaluated. Currently, there is inconclusive evidence about clinically important differences in children and adolescents who received eye movement desensitisation and reprocessing (EMDR) compared to wait-list controls on reducing PTSD symptom severity (Chemtob, Nakashima, & Hamada, 2002; Kemp, Drummond, & McDermott, 2010). A recent development is Narrative Exposure Therapy (NET), which promotes traumatic memory re-exposure, and reprocessing aided by the development of a chronological narrative. NET was successfully trialled in 31 Sri Lankan children aged 8–14 years exposed to the 2004 tsunami; six sessions of NET was compared to a similar number of meditation relaxation therapy sessions. There was a trend for a reduced likelihood of PTSD at 1 month and 6 month follow-up in the NET group (Catani et al., 2009). In another small study (*n*=21) with a child specific version (KIDNET), a trained therapist delivered 8×90–120 min sessions to refugee children who met diagnostic criteria for PTSD and had on average four different types of serious traumatic event exposure. Compared to wait-list controls, the KIDNET group demonstrated clinically significant improvements in symptoms and functioning (Ruf et al., 2010). See the NET review of Robjant and Fazel (2010) for further discussion.

Our TF-CBT intervention (Cobham & McDermott, 2011) closely follows dissemination of the results of school-based screening with the aim of commencing therapy 4–6 months post-disaster. Our model included offering local clinicians a 2-day training course in TF-CBT (Australian Centre for Posttraumatic Mental Health [ACPMH], 2009), as well as training in a manualised TF-CBT intervention and supervision during the delivery of this intervention. Our aim in training local clinicians is to leave a legacy of skilled TF-CBT practitioners once the disaster intervention is concluded. Therapy is conducted in the school setting which is consistent with many contemporary post-disaster approaches (Chemtob et al., 2002; Gelkopf & Berger, 2009; Salloum & Overstreet, 2008; Wolmer, Laor, & Yazgan, 2003). The intervention is a 10-session programme, influenced by the Ehlers—Clarke model of PTSD (Ehlers & Clarke, 2000). The first two sessions are with the parent or caregiver and focus on psycho-education, outlining the child intervention, predicting the possibility of some worsening of the child's symptom once the child begins his/her trauma narrative, and motivating the parent to be involved in the programme including homework and graded exposure tasks. Our emphasis on parent involvement is influenced by evidence that the addition of parent sessions to child CBT leads to improved long-term outcomes in children with anxiety (Cobham, Dadds, Spence, & McDermott, 2010). The eight child sessions initially focus on developing a shared vocabulary about thoughts, feelings, and behaviours, followed by telling and re-telling the trauma narrative. In the context of containing a therapeutic environment, this re-telling both facilitates habituation to the anxiety and distress associated with the traumatic event and allows the identification of “hot spot” thoughts about the experience. These unhelpful thoughts or beliefs then become targets of a cognitive intervention. Finally, behavioural tasks are developed, practiced, en-acted, and supervised; an example being returning to a school where a traumatic event occurred.

## Conclusion

Natural disasters will always occur. Advances in infrastructure design and construction, and technological aides to early warning may mitigate the perceived threat experienced by some children and adolescents, with some decrease in post-disaster mental health presentations. However, given the sudden unexpected nature of many disasters, these advances are unlikely to be a universal panacea. When considering future disasters it is plausible that existing services will continue to struggle to meet surge demands and children and adolescents who may benefit from early intervention will not receive such services. This places these individuals at risk of experiencing chronic symptoms and related family, peer, and/or school impairment. In response to lack of capacity, services require new processes to facilitate assessment and triage of children and adolescents. One way to achieve this is a stepped-care model that offers interventions at various levels along a continuum of care.

From the perspective of delivering a post-disaster stepped-care model, our team has the advantage of established resources, enabling interventions to be employed rapidly. However, we face significant challenges in being able to provide further empirical evidence for our model. For example, regarding level 1 interventions, research that quantifies the uptake of communication resources, the acceptability of the content, and the extent to which the content is retained and influences behaviour by consumers is required. How technology innovations including social media can improve the dissemination and reach of level 1 interventions is an important research area. For level 2 interventions, we need to better demonstrate trainees’ satisfaction with resources and their competency in delivering the intervention or programme in which they have been trained. Further, we need to better understand the end users’ response to resources and again whether it changes their knowledge and behaviour. We are currently evaluating parent satisfaction with the screening process. Other screening-related research includes the relative efficiency of screening very high risk, moderate and low risk communities with a view to better understanding whether screening is more efficacious as a universal or targeted process. Work is also underway looking at optimising what can be a lengthy questionnaire screening battery into a less burdensome screen that optimises sensitivity and specificity. Overall, model of care developments include possible steps within our three tiered system, for example will some individuals respond to a low-intensity TF-CBT intervention? All children and adolescents in our TF-CBT intervention completed the programme suggesting good participant acceptability of the approach and resources. However, can we employ technology that enhances the reach of the intervention without diminishing its effectiveness? Finally, our current model includes work with parents on post-disaster parenting. A related issue is whether a comprehensive child and adolescent stepped-care response can include the capacity to provide effective treatments for parents who themselves are suffering from a post-disaster mental health syndrome. Whilst further research is required, it is arguable that it is difficult for a child to recover (both symptomatic and a return to pre-disaster levels of functioning and quality of life) in the context of a parent who is experiencing a post-disaster mental health syndrome. Finally, the necessity of delivering a stepped-care model to enhance reach in the challenging post-disaster environment has the potential of informing how mainstream CAMHS services can potentially provide a range of treatments across levels of intensity and arrange these interventions in a stepped-care hierarchical approach.
